# Fabrication and characterization of La_2_Zr_2_O_7_ films on different buffer architectures for YBa_2_Cu_3_O_7−*δ*_ coated conductors by RF magnetron sputtering

**DOI:** 10.1186/1556-276X-8-109

**Published:** 2013-02-27

**Authors:** Da Xu, Linfei Liu, Guina Xiao, Yijie Li

**Affiliations:** 1 Research Center of Applied Superconductivity and Key Laboratory of Artificial Structures and Quantum Control (Ministry of Education), Department of Physics, Shanghai Jiao Tong University, 800 Dongchuan Road, Shanghai 200240, People’s Republic of China

**Keywords:** LZO, Buffer layer, YBCO, Texture

## Abstract

La_2_Zr_2_O_7_ (LZO) films were grown on different buffer architectures by radio frequency magnetron sputtering for the large-scale application of YBa_2_Cu_3_O_7−*x*_ (YBCO)-coated conductors. The three different buffer architectures were cerium oxide (CeO_2_), yttria-stabilized zirconia (YSZ)/CeO_2_, and CeO_2_/YSZ/CeO_2_. The microstructure and surface morphology of the LZO film were studied by X-ray diffraction, optical microscopy, field emission scanning electron microscopy, and atomic force microscopy. The LZO films prepared on the CeO_2_, YSZ/CeO_2_, and CeO_2_/YSZ/CeO_2_ buffer architectures were preferentially *c*-axis-oriented and highly textured. The in-plane texture of LZO film on CeO_2_ single-buffer architecture was ∆ *φ* = 5.5° and the out-of-plane texture was ∆ *ω* = 3.4°. All the LZO films had very smooth surfaces, but LZO films grown on YSZ/CeO_2_ and CeO_2_/YSZ/CeO_2_ buffer architectures had cracks. The highly textured LZO film grown on CeO_2_-seed buffered NiW tape was suitable for the epitaxial growth of YBCO film with high currents.

## Background

In the productive process of the second-generation high-temperature superconducting (HTS) strips, the epitaxial growth of highly textured buffer layer is important for the fabrication of YBa_2_Cu_3_O_7−*x*_ (YBCO) superconducting film [1]. Several technologies have been used to fabricate biaxially textured YBCO-coated conductors on metallic substrates, including inclined substrate deposition [2], ion beam-assisted deposition [3], and rolling-assisted biaxially textured substrate (RABiTS) [4]. Among them, the RABiTS approach appears to be one of the most promising routes for scale-up processing of the second-generation HTS strips due to its easily controlled buffer growth, highly textured substrates, and cost-effective processing techniques such as chemical solution deposition (CSD) [5-7]. A wide variety of oxide materials, such as cerium oxide (CeO_2_), yttria-stabilized zirconia (YSZ), yttrium oxide (Y_2_O_3_), and La_2_Zr_2_O_7_ (LZO), have been successfully used as potential buffer layers for the preparation of YBCO-coated conductor [8,9]. Among them, CeO_2_ (cubic, *a* = 5.41 Å, lattice mismatch CeO_2_/NiW = 8.2%, and YBCO/CeO_2_ = 0.52%) is a preferred and well-examined buffer layer that grows nicely due to its chemical stability and lattice match with the NiW substrate and YBCO superconducting layer [10]. Unfortunately, epitaxial CeO_2_ films crack extensively when the thickness of CeO_2_ film exceeds 100 nm. Therefore, a stack of CeO_2_/YSZ/CeO_2_ or CeO_2_/YSZ/Y_2_O_3_ is commonly used as an effective buffer architecture satisfying the epitaxial growth of YBCO-coated conductors.

LZO films have been applied effectively as a buffer layer for YBCO-coated conductors prepared by various methods. From the results of previous studies, Ying et al. reported that they prepared CeO_2_/LZO and single LZO buffer layers for YBCO films by pulsed laser deposition (PLD) [11,12]. Knoth et al. reported that they fabricated LZO buffer layer by CSD with the out-of-plane texture Δ*ω* = 7.2° and the in-plane texture Δ*φ* = 6.9° [13]. Wee et al. reported that they obtained LZO films by slot die coating of CSD with the out-of-plane texture of Δ*ω* = 5.7° and the in-plane texture of Δ*φ* = 6.7° [14]. However, the low texture and rough surface morphology of LZO film cannot satisfy the requirements of the epitaxial growth of high-performance YBCO film. Therefore, it is necessary to prepare an LZO film with high in-plane and out-of-plane textures and smooth surfaces in order to achieve an YBCO film with high critical current density (*J*_*c*_).

In the present work, we fabricate highly textured LZO films on the CeO_2_, YSZ/CeO_2_, and CeO_2_/YSZ/CeO_2_ buffered NiW tapes under optimal conditions by radio frequency (RF) magnetron sputtering. The microstructure and surface morphology of LZO film are investigated. YBCO-coated conductors are prepared on the LZO/CeO_2_, LZO/YSZ/CeO_2_, and LZO/CeO_2_/YSZ/CeO_2_ buffer architectures, and we also discuss the superconductivity of YBCO-coated conductors.

## Methods

Highly textured RABiTS tapes were used for the subsequent preparation of CeO_2_ seed layer, YSZ buffer layer, CeO_2_ cap layer, LZO film, and YBCO-coated conductor. The RABiTS tape was provided by evico magnetics GmbH in Dresden, Germany [15]. The in-plane and out-of-plane textures of RABiTS tape used in this study were evaluated by the full width at half maximum (FWHM) of the *φ*-scan and *ω*-scan as ∆ *φ* = 6° to 7° and ∆ *ω* = 5° to 6°, respectively. The RABiTS tape was approximately 80 μm in thickness, and the average roughness value of surface roughness was less than 5 nm. A long RABiTS tape was cut into several short samples, which were 10 cm in length and 10 mm in width. Before the preparation of LZO film, all the CeO_2_ seed layer, YSZ buffer layer, and CeO_2_ cap layer were fabricated on these short samples by PLD. A KrF excimer laser (LPX220, Lambda Physik Inc., Fort Lauderdale, FL, USA) with a wavelength of 248 nm was used for CeO_2_, YSZ, and YBCO film deposition, and the incident angle between the laser beam and the target surface was 45°. Detailed experiments were reported in other works [16,17]. From previous experiments [16], we obtained the samples of CeO_2_, YSZ/CeO_2_, and CeO_2_/YSZ/CeO_2_ buffered NiW tapes. We then fabricated LZO films on the CeO_2_, YSZ/CeO_2_, and CeO_2_/YSZ/CeO_2_ buffered NiW tapes by RF magnetron sputtering in Ar gas of 20 sccm at a substrate temperature of 600°C. Deposition pressure and applied RF power were fixed at 20 Pa and 100 W, respectively. The distance between the target and the substrate was 5 cm. Finally, we fabricated the YBCO films on the LZO/CeO_2_, LZO/YSZ/CeO_2_, and LZO/CeO_2_/YSZ/CeO_2_ buffer architectures at the substrate temperature of 800°C by PLD. The oxygen partial pressure was 50 Pa. The laser energy was 200 mJ, and the laser repetition rate was 50 Hz. After deposition, YBCO films were quickly cooled to room temperature and then annealed at 500°C in pure O_2_ gas for 1 h. More details can be found elsewhere [18,19].

The structure and texture of LZO film were measured by a general area detector diffraction system (D8 Discover with GADDS, Bruker AXS, Inc., Fitchburg, WI, USA) with Cu-K*α* radiation operated at 40 mA and 40 kV. The surface morphologies of LZO films were observed by optical microscopy (OM, BX51M, Olympus Corporation, Shinjuku-ku, Japan), high-resolution field emission scanning electronic microscopy (FEI Sirion 200, FEI Company, Hillsboro, OR, USA) operated at 5 kV, and tapping mode atomic force microscopy (AFM, Multimode 8, Bruker AXS, Inc., Fitchburg, WI, USA). The critical current (*I*_*c*_) of YBCO-coated conductor was evaluated by the conventional four-probe method at 77 K and self field using a criterion of 1 μV/cm.

## Results and discussion

**Figure 1:**
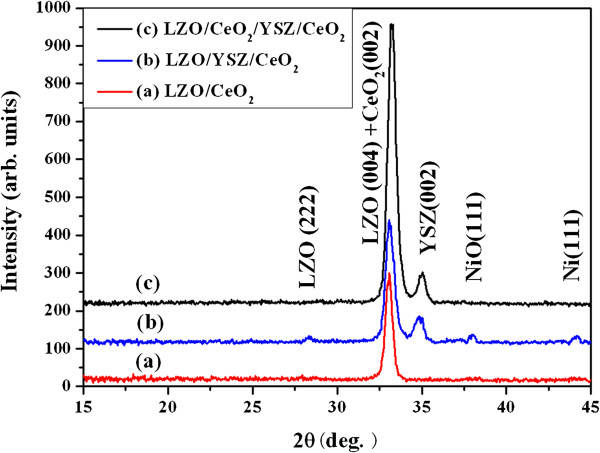
**XRD *θ*-2*θ *scans of LZO films prepared on three different buffered NiW tapes.** The three different buffer architectures are curves (**a**) CeO_2_, (**b**) YSZ/CeO_2_, and (**c**) CeO_2_/YSZ/CeO_2_.

**Figure 2:**
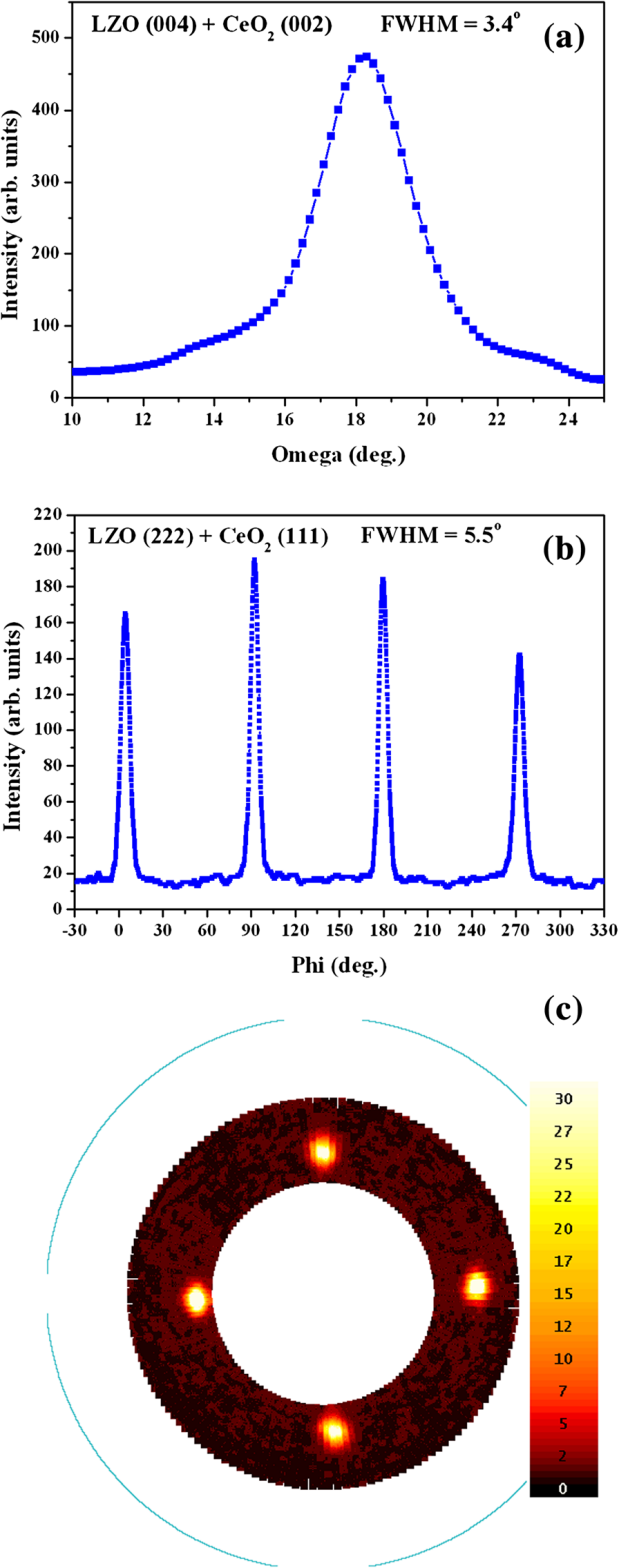
**Typical XRD patterns of LZO films.** (**a**) *ω*-scan pattern, (**b**) *φ*-scan pattern, and (**c**) pole figure of LZO films grown on CeO_2_ buffered NiW tapes with the texture of ∆ *ω* = 3.4° and ∆ *φ* = 5.5°.

To avoid the thickness effect, LZO films of the same thickness were fabricated on CeO_2_, YSZ/CeO_2_, and CeO_2_/YSZ/CeO_2_ buffered NiW substrates by RF magnetron sputtering under optimal conditions. X-ray diffraction (XRD) *θ*-2*θ* scans of LZO films are carried out to characterize the structure of LZO films, as shown in Figure 1. LZO (004) peak and CeO_2_ (002) peak are at the same 2*θ* position. The LZO film grown on CeO_2_-seed and CeO_2_/YSZ/CeO_2_ buffered NiW tapes shows pure *c*-axis orientation as only (004) reflection of the LZO film, and no LZO (222) peak is observed. This indicates that LZO film is preferentially oriented with the *c*-axis perpendicular to the substrate surface and has an excellent crystallinity. However, small LZO (222) peak is detected in the LZO sample grown on YSZ/CeO_2_ buffered NiW tape, which resulted from the minority misoriented grains in LZO films. These misoriented grains are grown on top of randomly oriented grains in the NiW substrate or formed by coalesced larger droplets. The out-of-plane and in-plane epitaxial orientations of LZO films are confirmed using *ω*-scan and *φ*-scan XRD measurements. Table 1 shows out-of-plane and in-plane textures of LZO films grown on three different buffered NiW tapes. From the texture analysis data, it can be seen that the LZO film prepared on the CeO_2_-seed buffered NiW tape has the best out-of-plane texture of ∆ *ω* = 3.4° and the in-plane texture of ∆ *φ* = 5.5°. The out-of-plane texture and in-plane texture of the YSZ buffer layer are ∆ *ω* = 4.2° and ∆ *φ* = 7.2°, respectively. The rocking curves and pole figure of the LZO film fabricated on the CeO_2_-seed buffered NiW tape are shown in Figure 2. The FWHM values of both *ω*-scan and *φ*-scan rocking curves of LZO film on the CeO_2_-seed buffered NiW tape are ∆ *ω* = 3.4° in Figure 2a and ∆ *φ* = 5.5° in Figure 2b. This indicates that LZO film is preferentially *c*-axis-oriented and has excellent high out-of-plane and in-plane alignments. In Figure 2c, the fourfold symmetry in the LZO pole figure indicates a single cube-textured LZO film.

**Table 1:** Texture analysis data of LZO films grown on three different buffer architectures

	**Out-of-plane texture ∆ *ω *(deg)**		**In-plane texture ∆ *φ *(deg)**	
	**LZO (004) + CeO**_**2 **_**(002)**	**YSZ (002)**	**LZO (222) + CeO**_**2 **_**(111)**	**YSZ (111)**
LZO/CeO_2_/NiW	3.4		5.5	
LZO/YSZ/CeO_2_/NiW	3.8	4.2	6.0	7.2
LZO/CeO_2_/YSZ/CeO_2_/NiW	3.5	4.2	6.1	7.2

**Figure 3:**
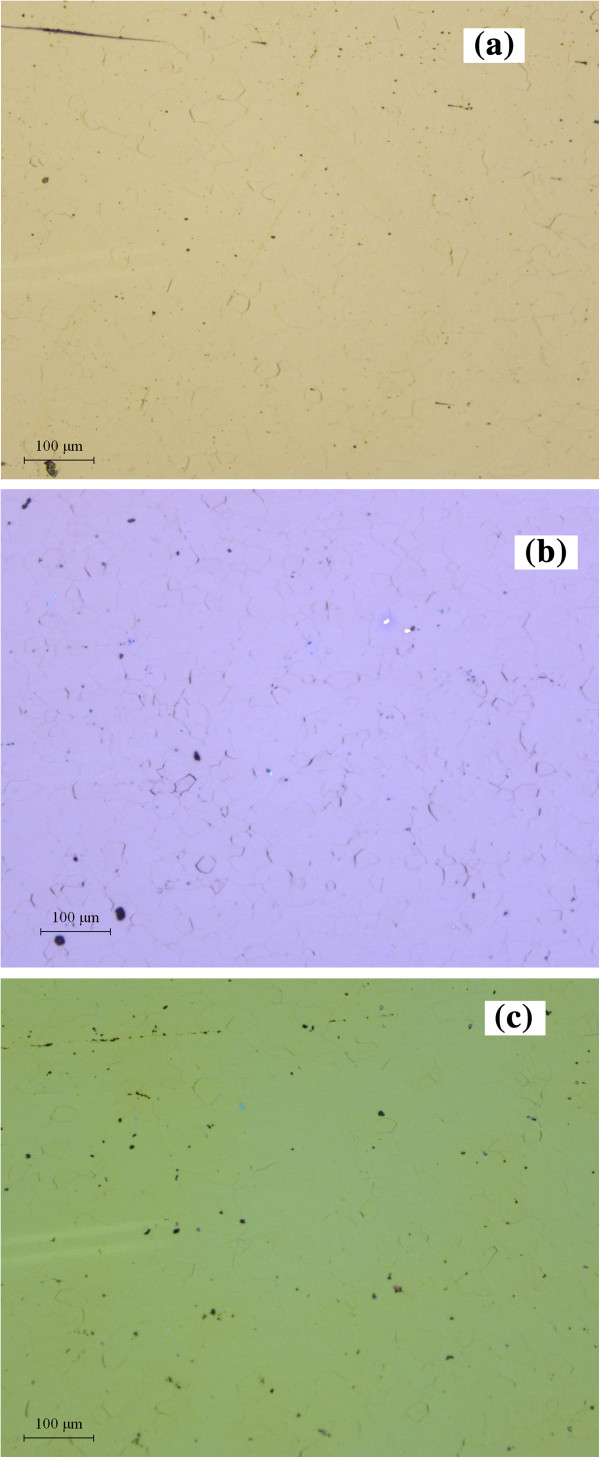
**Optical photographs of LZO films.** Prepared on three buffer architectures of (**a**) CeO_2_, (**b**) YSZ/CeO_2_, and (**c**) CeO_2_/YSZ/CeO_2_.

**Figure 4:**
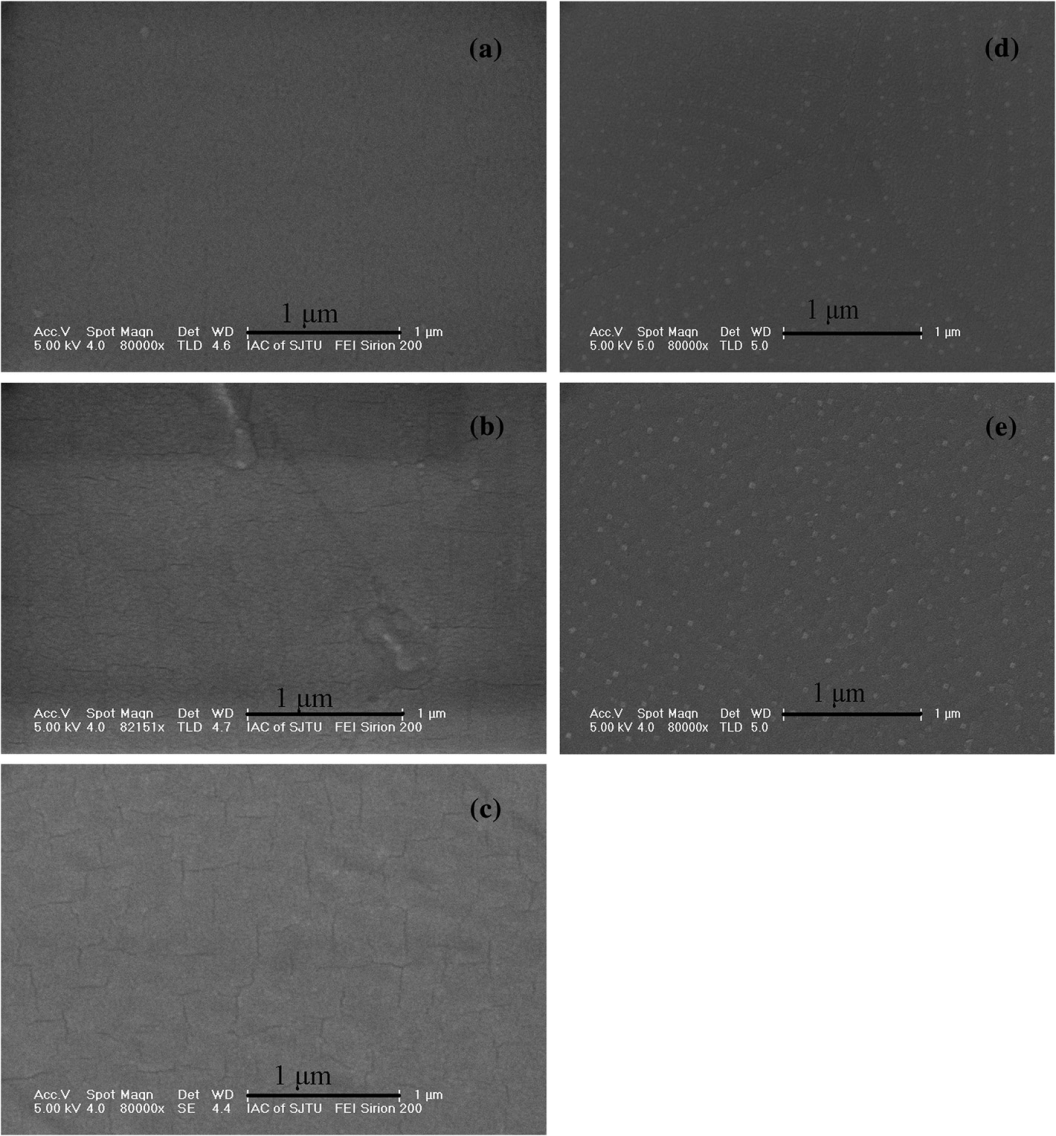
**SEM images of LZO films.** Fabricated on the (**a**) CeO_2_, (**b**) YSZ/CeO_2_, and (**c**) CeO_2_/YSZ/CeO_2_ buffered NiW tapes**.** (**d**) and (**e**) are SEM images of LZO films grown on YSZ/CeO_2_ and CeO_2_/YSZ/CeO_2_ buffer architectures with the thickness of the buffer layer less than the critical value, respectively.

**Figure 5:**
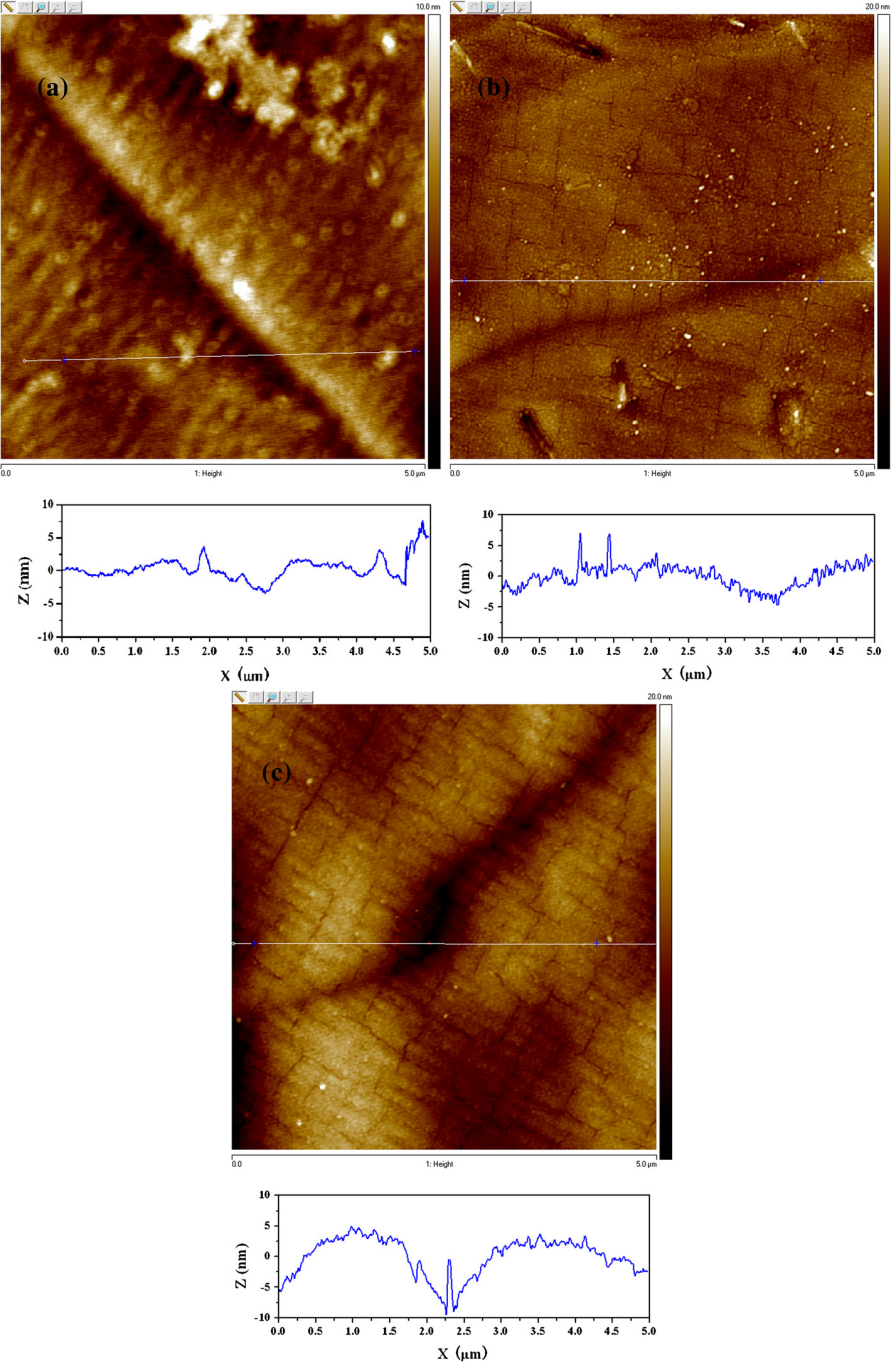
**AFM photographs of LZO films.** Grown on the (**a**) CeO_2_, (**b**) YSZ/CeO_2_, and (**c**) CeO_2_/YSZ/CeO_2_ buffer architectures.

xTo investigate the films deeply and broadly, the surface morphologies of LZO films fabricated on CeO_2_, CeO_2_/YSZ, and CeO_2_/YSZ/CeO_2_ buffered NiW tapes are observed by OM, SEM, and AFM. From optical photographs shown in Figure 3, it is demonstrated that the surface of all LZO films on CeO_2_, CeO_2_/YSZ, and CeO_2_/YSZ/CeO_2_ buffered NiW tapes are all flat without any island or particle in the area of 1 mm × 1 mm. Only a few grain boundaries are observed in the surfaces of LZO films. The surfaces of LZO films are flat enough in a large area with only shallow grain boundaries. This indicates that LZO buffer layers are suitable for the sequential epitaxial growth of YBCO films. In Figure 4, SEM images also indicate that all the LZO films deposited on three different buffer architectures have excellent smooth surface. Figure 4a shows that the LZO film grown on CeO_2_ seed layer has no microcrack and is flat without any island in the area of 3 μm × 4 μm. However, in Figure 4b,c, microcracks are observed in LZO films grown on YSZ/CeO_2_ and CeO_2_/YSZ/CeO_2_ buffered NiW tapes, which resulted from the film structural stress when the thickness of the entire buffer layer exceeds the critical value. The thicknesses of CeO_2_ seed layer, YSZ buffer layer, and CeO_2_ cap layer are 50, 100, and 200 nm, respectively. The thickness of the LZO buffer layer grown on single CeO_2_, YSZ/CeO_2_, and CeO_2_/YSZ/CeO_2_ buffered NiW substrates are the same which is 100 nm. When the thicknesses of all buffer layers exceed the critical value of 200 nm, cracks appear in LZO films grown on the YSZ/CeO_2_ and CeO_2_/YSZ/CeO_2_ buffer architectures. LZO films grown on YSZ/CeO_2_ and CeO_2_/YSZ/CeO_2_ buffer architectures with the thickness of the buffer layer less than the critical value are shown in Figure 4d,e, respectively. From the pictures of Figure 4d,e, it is clear that LZO films have no microcracks, but small particles on the surfaces have the number density of 30/μm^2^. Tapping mode AFM images in Figure 5 illustrated that the root mean square (RMS) surface roughness of LZO films grown on CeO_2_-seed, YSZ/CeO_2_, and CeO_2_/YSZ/CeO_2_ buffer architectures were 1.2, 1.9, and 2.5 nm in the scanning area of 5 μm × 5 μm. The surface of the LZO film becomes much rougher when the thickness of the entire buffer layer is increased. The grain size of particles on the surface of the LZO film is about 0.2 μm in diameter. The grain-boundary depths of LZO films prepared on CeO_2_-seed, YSZ/CeO_2_, and CeO_2_/YSZ/CeO_2_ buffer architectures are about 10 nm, and the grain-boundary widths are approximately 1 μm. These results indicate that LZO films grown on the CeO_2_-seed, YSZ/CeO_2_, and CeO_2_/YSZ/CeO_2_ buffer architectures are indeed high quality. Figure 5a shows the LZO film grown on CeO_2_ seed layer is flat and dense with no cracks. In Figure 5b,c, LZO films grown on the YSZ/CeO_2_ and CeO_2_/YSZ/CeO_2_ buffer architectures are also flat and dense but are cracked. These results are corresponding with the results of SEM observations. The cracks in LZO film will give rise to decrease in *J*_*c*_ of upper YBCO superconducting layer.

**Figure 6:**
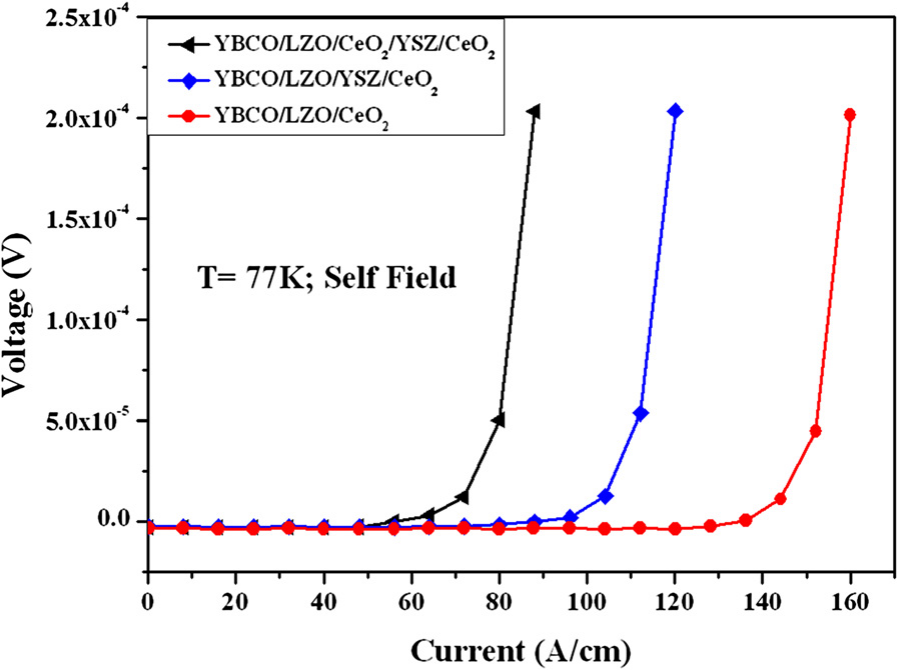
**End-to-end voltage–current characteristics of YBCO-coated conductors.** Deposited on the LZO/CeO_2_, LZO/YSZ/CeO_2_, and LZO/CeO_2_/YSZ/CeO_2_ buffered NiW tapes using the conventional four-probe method tested at 77 K and self field.

To verify whether LZO buffer layer was suitable for the epitaxial growth of YBCO superconducting film, YBCO-coated conductors were deposited on highly textured LZO/CeO_2_, LZO/YSZ/CeO_2_, and LZO/CeO_2_/YSZ/CeO_2_ buffer architectures. The *I*_*c*_ of YBCO films on the LZO/CeO_2_, LZO/YSZ/CeO_2_, and LZO/CeO_2_/YSZ/CeO_2_ buffer architectures were measured at 77 K and self field by the conventional four-probe method without microbridge patterning shown in Figure 6. The critical current density was calculated from *J*_*c*_ = *I*_*c*_/(*a* × *b*) (*a* and *b* are the film width and thickness in centimeters, respectively). From the voltage–current characteristic curves, the *I*_*c*_ of YBCO films were recorded by using the criterion of 1 μV/cm. Figure 6 shows that the *I*_*c*_ of YBCO films grown on the LZO/CeO_2_, LZO/YSZ/CeO_2_, and LZO/CeO_2_/YSZ/CeO_2_ buffer architectures are 140, 100, and 60 A/cm, respectively. The thicknesses of YBCO films grown on the LZO/CeO_2_, LZO/YSZ/CeO_2_, and LZO/CeO_2_/YSZ/CeO_2_ buffer architectures are all the same which is 500 nm. As expected, the highest *J*_*c*_ of 2.8 MA/cm^2^ at 77 K, self field is obtained for YBCO-coated conductor grown on LZO/CeO_2_ buffered NiW tape. Therefore, the highly textured LZO film grown on CeO_2_-seed buffered NiW tape, which has smooth surface without any island and crack, is suitable for the epitaxial growth of high-performance YBCO-coated conductors.

## Conclusions

LZO films were grown on CeO_2_, YSZ/CeO_2_, and CeO_2_/YSZ/CeO_2_ buffered RABiTS tapes by RF magnetron sputtering. As a result, LZO films prepared on the single CeO_2_ and CeO_2_/YSZ/CeO_2_ buffer architectures were preferentially *c*-axis-oriented and highly textured. Only small LZO (222) peak was observed in the LZO film fabricated on YSZ/CeO_2_ buffered NiW tape. Both in-plane and out-of-plane textures of LZO film on the CeO_2_-seed buffered NiW tape were ∆ *φ* = 5.5° and ∆ *ω* = 3.4°. LZO films had very smooth surfaces, but microcracks were observed in LZO films grown on the YSZ/CeO_2_ and CeO_2_/YSZ/CeO_2_ buffer architectures. From the results discussed above, LZO film on CeO_2_-seed buffered NiW tape had the smoothest surface with the smallest RMS value and best in-plane and out-of-plane textures. The highly textured LZO film grown on CeO_2_-seed layer with smooth surface satisfied the requirements of epitaxial growth of YBCO-coated conductors with high currents.

## Abbreviations

AFM: Atomic force microscopy;CeO2: cerium oxide;CSD: chemical solution deposition;FWHM: full width at half maximum;HTS: high-temperature superconducting;Ic: critical current;Jc: critical current density;LZO: La_2_Zr_2_O_7_;OM: optical microscopy;PLD: pulsed laser deposition;RABiTS: rolling assisted biaxially textured substrate;RF: radio frequency;RMS: root mean square;XRD: X-ray diffraction;YBCO: YBa_2_Cu_3_O_7−*δ*_;YSZ: yttria-stabilized zirconia;Y2O3: yttrium oxide

## Competing interests

The authors declare that they have no competing interests.

## Authors’ contributions

DX participated in the design of the study, carried out the fabrication of LZO films, performed the statistical analysis, as well as drafted the manuscript. LL participated in the design of the study, carried out the preparation of NiW tapes with different buffer architectures, and revised the manuscript. GX helped to operate the RF magnetron sputtering system. YL participated in the design of the study, provided the theoretical and experimental guidance, and revised the manuscript. All authors read and approved the final manuscript.
